# Association of polycythemia with outcomes of acute decompensated heart failure: A matched and weighted cohort analysis

**DOI:** 10.1371/journal.pone.0345255

**Published:** 2026-04-20

**Authors:** Snir Perets, Sa’ar Minha, Shiri L. Maymon, Eran Kalmanovich, Gil Moravsky, Ido Minha, Avishay Grupper, Shmuel Fuchs, Gil Marcus

**Affiliations:** 1 Department of Cardiology, Shamir Medical Center, Be’er Ya’akov, Israel; 2 Gray Faculty of Medical and Health Sciences, Tel-Aviv University, Tel-Aviv, Israel; 3 Department of Otolaryngology, Tel-Aviv Sourasky Medical Center, Tel-Aviv, Israel; 4 The Rachel and Selim Benin School of Computer Science and Engineering, The Hebrew University of Jerusalem, Jerusalem, Israel; Saud Al-Babtain Cardiac Centre, SAUDI ARABIA

## Abstract

**Aims:**

The prognostic significance of polycythemia in acute decompensated heart failure (ADHF) is unclear. This study aimed to evaluate the clinical profile and prognostic implications of polycythemia compared with anemia and normocythemia in patients hospitalized with ADHF.

**Materials and methods:**

We retrospectively analyzed adult patients hospitalized with ADHF between 2007 and 2017. Patients were categorized by hemoglobin according to World Health Organization criteria: anemic (<13 g/dL men, < 12 g/dL women), normocythemic, or polycythemic (>18.5 g/dL men, > 16.5 g/dL women). Mahalanobis distance matching (MDM; 1:3:3) balanced baseline characteristics, with outcomes compared for in-hospital mortality, 30-day readmission, and long-term survival. Entropy balancing (EBAL) served as sensitivity analysis in the full cohort.

**Results:**

Of 8,332 patients, 5,615 (67.4%) were anemic, 2,639 (31.7%) normocythemic, and 78 (0.9%) polycythemic. Polycythemic patients were younger, predominantly male, and more likely to undergo coronary interventions and receive cardioprotective discharge medications. In the matched cohort (N = 546; 234 anemic, 234 normocythemic, 78 polycythemic), in-hospital mortality rates were similar (5.6%, 3.8%, 7.7%; p = 0.381). One-year mortality was highest in anemia (27.4%) vs. normocythemia (17.5%) and polycythemia (19.2%; p = 0.030). Five-year Kaplan–Meier survival was poorest in anemia, with overlapping curves for polycythemia and normocythemia (log-rank p = 0.027). Cox analysis (reference = normocythemia) showed higher mortality with anemia (HR 1.30, 95% CI 1.03–1.63) but not polycythemia (HR 0.90, 95% CI 0.64–1.27). Post-hoc pairwise log-rank tests (Bonferroni-corrected) confirmed no difference between polycythemia and normocythemia. EBAL-weighted analysis yielded consistent results (log-rank p < 0.001; anemia HR 1.76, 95% CI 1.61–1.92; polycythemia HR 1.14, 95% CI 0.83–1.57).

**Conclusions:**

Polycythemia is rare in hospitalized ADHF and, unlike anemia, is not associated with adverse short- or long-term outcomes.

## Introduction

Heart failure (HF) remains a leading cause of hospitalization, morbidity, and mortality worldwide, with prevalence rising steeply in older adults [1,2]. Prognosis in HF is shaped not only by cardiac dysfunction but also by comorbidities that modify risk and therapeutic response, including chronic kidney disease (CKD), diabetes mellitus (DM), chronic obstructive pulmonary disease (COPD), and anemia [3–6]. Among these, anemia has been extensively characterized and consistently associated with worse outcomes in both chronic HF and acute decompensated HF (ADHF), and contemporary guidelines emphasize evaluation and treatment of iron deficiency as part of comprehensive HF care [7–11].

In contrast, elevated hemoglobin (polycythemia) is less frequently discussed in HF. Polycythemia may reflect a primary myeloproliferative process or, more commonly, secondary adaptation to hypoxemia, smoking, or pulmonary disease. In acute coronary syndrome (ACS), polycythemia has been recently linked to adverse outcomes, plausibly through increased blood viscosity and thrombotic potential [12]. Whether a similar association exists for ADHF, which is related to ACS through mechanism as well as through common comorbidities – remains uncertain.

To address this gap in knowledge, we evaluated the clinical profile and prognostic implications of polycythemia in patients hospitalized with ADHF. We compared patients with polycythemia to those with normocythemia and anemia, for association with short- and long-term outcomes, in a large cohort of ADHF patients with detailed admission information and long-term follow-up.

## Materials and methods

### Data source

This retrospective cohort study was conducted at Shamir Medical Center and included adult patients admitted with ADHF between January 1, 2007, and December 31, 2017. Eligible patients were adults aged 18 years or older who received a primary diagnosis of ADHF at admission, as identified by ICD-9 codes 428.xx, 429.xx, and 514, and whose diagnosis was confirmed upon discharge. This study received ethical clearance from the Shamir Medical Center Institutional Review Board (IRB #ASF-0063-18). Initial authorization was secured on April 23, 2018, with the latest renewal granted on March 25, 2025. Because this was a retrospective analysis using de-identified records, the IRB waived the requirement for participant informed consent. Researchers first accessed the data for analysis on April 13, 2025. While the primary dataset contained personal identifiers, access was strictly limited to authorized physician investigators. All published findings are presented as aggregated data; furthermore, the results contain no small cells or other identifying characteristics that could potentially compromise patient anonymity.

### Study design

Patients were categorized into hemoglobin groups according to World Health Organization (WHO) criteria. Anemia was defined as hemoglobin <13 g/dL in men and <12 g/dL in women. Polycythemia was defined as hemoglobin >18.5 g/dL in men and >16.5 g/dL in women. Patients not meeting these thresholds were classified as normocythemic [13].

An initial review of the dataset revealed a relatively small number of patients with polycythemia. Consequently, we adopted a matched case-control design, applying Mahalanobis distance matching (MDM) which has been shown to achieve good balance in cohorts with small or rare exposure groups [14]. This matched cohort was used for the main outcomes analysis, which included in-hospital mortality, 30-day readmission, and long-term 1- and 5-year survival. In addition, a sensitivity 5-year survival analysis was conducted using the full original cohort, applying weighting by entropy balancing (EBAL) to achieve exact covariate balance between groups [15].

### Mahalanobis distance matching

MDM was used to construct a matched cohort in a 1:3:3 ratio, comprising patients with polycythemia, normocythemia, and anemia, respectively. Matching was performed without replacement to ensure unique control subjects. Covariates included in the matching procedure were chosen based on their established associations with heart failure outcomes: [16] age and sex [17], CKD [18], DM [19], ischemic heart disease (IHD) [20], atrial fibrillation (AF) [21], COPD [22], peripheral vascular disease (PVD) [23], and hypertension (HTN) [24,25]. Variables without consistent evidence of independent adverse prognostic significance in hospitalized heart failure populations, such as smoking status, were not included; additionally, the strong association of smoking with both the exposure (polycythemia) and an existing covariate (COPD) risked destabilizing the matching in the context of a small exposure group. Balance was evaluated using standardized mean differences (SMDs), with values <0.1 considered indicative of adequate adjustment.

### Entropy balancing – sensitivity analysis

For the full-cohort sensitivity analysis, weighting by EBAL was applied, targeting the polycythemia group. EBAL reweights patients in the anemia and normocythemia groups so that their weighted covariate distribution exactly matches that of the polycythemia group. The same covariates used for matching were specified in the EBAL optimization, with the squared term of age added to additionally balance variance in the age distribution. Weights were constrained to remain positive and normalized within groups to have mean 1. SMDs were calculated before and after weighting to evaluate covariate balance, with values <0.1 considered acceptable.

### Statistical analysis

Baseline characteristics were analyzed using univariate methods. Categorical variables are presented as frequencies and percentages and were compared using the chi-square test. Continuous variables are reported as means and standard deviations (SD) if normally distributed, or medians and interquartile ranges (IQR) if non-normally distributed. The Kolmogorov–Smirnov test was used to assess normality. Between-group comparisons were performed using Student’s t-test for normally distributed variables and the Kruskal–Wallis test for non-normally distributed variables. Survival analyses were conducted using Kaplan–Meier curves, and differences between groups were evaluated with the log-rank test.

Multivariable analyses for adjustment were not conducted as adjustment was achieved by the matching and weighting in both the primary and the sensitivity analyses.

## Results

### Baseline characteristics – full cohort

Baseline characteristics are shown in Table 1. The cohort included 8,332 patients hospitalized with ADHF, of whom 5,615 (67.4%) had anemia, 2,639 (31.7%) had normocythemia, and 78 (0.9%) had polycythemia. Patients with polycythemia were significantly younger, with a mean age of 67.5 ± 13.3 years, compared to 74.4 ± 12.9 years in the normocythemia group and 77.3 ± 11.5 years in the anemia group (p < 0.001). Female sex was less common in the polycythemia group (25.6%) than in the normocythemia (54.1%) and anemia groups (47.7%) (p < 0.001). Renal failure and ischemic heart disease were more frequent among anemic patients (39.3% and 42.3%, respectively) compared with normocythemia and polycythemia (p < 0.001 for both).

**Table 1:** Baseline characteristics of the full cohort, stratified by hemoglobin groups.

	Anemia (N = 5615)	Normocythemia (N = 2639)	Polycythemia (N = 78)	p-value
Female sex, n (%)	2677.0 (47.7)	1427.0 (54.1)	20.0 (25.6)	<0.001
Age, mean±SD	77.3 ± 11.5	74.4 ± 12.9	67.5 ± 13.3	<0.001
Medical history, n (%)				
Ischemic heart disease	2374 (42.3)	946 (35.8)	30 (38.5)	<0.001
Renal failure	2206 (39.3)	520 (19.7)	16 (20.5)	<0.001
Atrial fibrillation	1773 (31.6)	779 (29.5)	18 (23.1)	0.055
Hypertension	981 (17.5)	427 (16.2)	13 (16.7)	0.346
Diabetes mellitus	3038 (54.1)	1081 (41.0)	36 (46.2)	<0.001
COPD	890 (15.9)	398 (15.1)	17 (21.8)	0.218
Peripheral vascular disease	472 (8.4)	126 (4.8)	4.0 (5.1)	<0.001
Obesity	1140 (20.3)	592 (22.4)	23.0 (29.5)	0.016
Smoking	797 (14.2)	505 (19.1)	25.0 (32.1)	<0.001
Chronic medications, n (%)				
Digoxin	286 (5.1)	142 (5.4)	2 (2.6)	0.5
Diuretics	3179 (56.6)	1367 (51.8)	37 (47.4)	<0.001
Alpha blockers	733 (13.1)	159 (6.0)	1 (1.3)	<0.001
Beta blockers	2061 (36.7)	930 (35.2)	27 (34.6)	0.416
Calcium channel blockers	1536 (27.4)	572 (21.7)	11 (14.1)	<0.001
ACEI	1054 (18.8)	502 (19.0)	12 (15.4)	0.711
ARBs	672 (12.0)	247 (9.4)	6 (7.7)	0.001
Spironolactone	156 (2.8)	47 (1.8)	2 (2.6)	0.024
Anti arrhythmic	387 (6.9)	177 (6.7)	2 (2.6)	0.313
Anti platelets	2431 (43.3)	1091 (41.3)	34 (43.6)	0.243
Oral anticoagulation	788 (14.0)	381 (14.4)	10 (12.8)	0.837
Statins	2232 (39.8)	1021 (38.7)	27 (34.6)	0.451
Laboratory indices, median [IQR]				
WBC, K/µL	8.8 [6.8-11.7]	9.9 [7.8-13.0]	12.1 [9.2-15.2]	<0.001
Hemoglobin, g/dL	10.8 [9.7-11.6]	13.5 [12.9-14.4]	16.9 [16.7-17.6]	<0.001
Urea, mg/dL	57.2 [40.2-86.6]	42.3 [33.0-57.6]	39.7 [30.4-54.1]	<0.001
Sodium, mmol/L	138.0 [134.0-141.0]	138.0 [135.5-141.0]	139.0 [136.0-141.0]	<0.001
Creatinine, mg/dL	1.2 [0.9-1.8]	1.0 [0.8-1.2]	1.2 [0.8-1.3]	<0.001
Left Ventricular Ejection Fraction by echocardiography, n (%)*				0.002
Preserved (EF ≥ 50%)	804.0 (49.5)	397.0 (43.2)	8.0 (25.8)	
Mildly reduced (EF = 40–49%)	247.0 (15.2)	131.0 (14.3)	6.0 (19.4)	
Moderately reduced (EF = 30–39%)	397.0 (24.4)	260.0 (28.3)	13.0 (41.9)	
Severely reduced (EF < 30%)	177.0 (10.9)	130.0 (14.2)	4.0 (12.9)	

ACEI, angiotensin-converting enzyme inhibitor; ARB, angiotensin receptor blocker; COPD, chronic obstructive pulmonary disease; EF, ejection fraction; IQR, interquartile range; SD, standard deviation; WBC, white blood cell count.

* Echocardiographic data were available for 2,574 patients (30.9% of the cohort).

Index admission interventions and discharge medications are summarized in Table 2. Coronary procedures were more common in polycythemic, with 17.9% undergoing angiography and 24.4% PCI, compared with 7.5% and 14.1% in normocythemic and 3.7% and 6.6% in anemic patients (both p < 0.001); coronary artery bypass grafting (CABG) was also more frequent in polycythemic (6.4% vs. 3.0% and 1.7%, p < 0.001), while dialysis occurred mainly in anemic patients (1.8% vs. 0.5% and 0%, p < 0.001). At discharge, diuretics were prescribed most often in anemic (75.2%), intermediate in normocythemic (71.6%), and least in polycythemic patients (64.1%, p < 0.001). In contrast, angiotensin converting enzyme inhibitors, beta receptor blockers, and antiplatelets were more frequently prescribed in polycythemic (35.9%, 64.1%, and 70.5%) than in anemic (25.7%, 53.1%, and 57.0%) or normocythemic patients (32.7%, 55.7%, and 61.4%) (all p ≤ 0.016), while oral anticoagulants were less frequently prescribed (20.5% vs. 25.1% and 27.7%, p = 0.024).

**Table 2:** Index admission interventions and discharge medications in the full cohort, stratified by hemoglobin group.

	Anemia (N = 5,615)	Normocythemia (N = 2,639)	Polycythemia (N = 78)	p-value
Index admission interventions, n (%)				
Pacemaker implantation	69 (1.2)	32 (1.2)	1 (1.3)	0.997
CRT implantation	14 (0.2)	7 (0.3)	0 (0.0)	0.897
Dialysis	102 (1.8)	13 (0.5)	0 (0.0)	<0.001
Coronary angiography	208 (3.7)	198 (7.5)	14 (17.9)	<0.001
PCI	369 (6.6)	371 (14.1)	19 (24.4)	<0.001
CABG	93 (1.7)	79 (3.0)	5 (6.4)	<0.001
Discharge medications, n (%)				
Digoxin	227 (4.0)	143 (5.4)	1 (1.3)	0.007
Diuretics	4,220 (75.2)	1,890 (71.6)	50 (64.1)	<0.001
Alpha blockers	959 (17.1)	235 (8.9)	3 (3.8)	<0.001
Beta blockers	2,980 (53.1)	1,470 (55.7)	50 (64.1)	0.016
Calcium channel blockers	1,903 (33.9)	727 (27.5)	17 (21.8)	<0.001
ACEI	1,443 (25.7)	862 (32.7)	28 (35.9)	<0.001
ARBs	819 (14.6)	357 (13.5)	6 (7.7)	0.112
Spironolactone	156 (2.8)	63 (2.4)	1 (1.3)	0.442
Antiarrhythmic	516 (9.2)	249 (9.4)	9 (11.5)	0.74
Antiplatelets	3,203 (57.0)	1,620 (61.4)	55 (70.5)	<0.001
Oral anticoagulants	1,408 (25.1)	730 (27.7)	16 (20.5)	0.024
Statins	3,023 (53.8)	1,533 (58.1)	44 (56.4)	0.001

ACEI, angiotensin-converting enzyme inhibitor; ARB, angiotensin receptor blocker; CABG, coronary artery bypass grafting; CRT, cardiac resynchronization therapy; PCI, percutaneous coronary intervention.

### Covariate balance after matching and weighting

**Fig 1:**
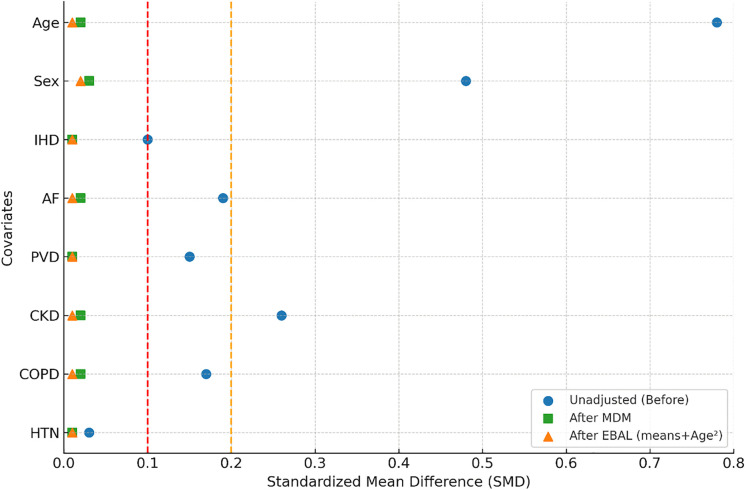
Covariate balance before and after adjustment. Standardized mean differences (SMDs) are shown for each covariate in the original unmatched cohort (blue circles), after Mahalanobis distance matching (MDM; green squares), and after entropy balancing (EBAL; orange triangles). Vertical dashed lines mark SMD thresholds of 0.1 (red) and 0.2 (orange). CKD, chronic kidney disease; COPD, chronic obstructive pulmonary disease; AF, atrial fibrillation; IHD, ischemic heart disease; PVD, peripheral vascular disease; HTN, hypertension.

Covariate balance before and after adjustment is shown in Fig 1. MDM markedly improved balance in baseline characteristics, with all post-matching SMDs < 0.1. EBAL weighting achieved similar results in the full cohort, with all post-weighting SMDs essentially reduced to zero by design. These findings confirm that both approaches successfully created balanced pseudo-randomized cohorts suitable for outcome comparison.

### Clinical outcomes – matched Cohort

Clinical outcomes are summarized in Table 3. In-hospital mortality was 5.6% in the anemia group, 3.8% in normocythemia, and 7.7% in polycythemia (n = 6), with no statistically significant difference (p = 0.381). At one year, mortality was highest among patients with anemia (27.4%), compared with 17.5% normocythemic and 19.2% in polycythemic patients (p = 0.030). Post-hoc pairwise comparisons with Bonferroni correction confirmed that this difference was driven by higher mortality in the anemia group compared with normocythemia (p = 0.044), whereas polycythemia and normocythemia did not differ (p = 1.000). The 30-day readmission rate was lower in the polycythemia group (9.6%) compared with the anemia and normocythemia groups (20.6%), although this difference did not meet the threshold for statistical significance (p = 0.084). Median length of stay was similar across groups (6–7 days, p = 0.734).

**Table 3:** Clinical outcomes in the matched cohort (Mahalanobis distance matching, 3:3:1), stratified by hemoglobin group.

Outcome, n (%)	Anemia (N = 234)	Normocythemia (N = 234)	Polycythemia (N = 78)	p-value
Length of stay, days, mean ± SD	6.4 ± 6.1	6.9 ± 6.6	6.8 ± 6.5	0.734
In-hospital mortality	13.0 (5.6)	9.0 (3.8)	6.0 (7.7)	0.381
In-Hospital Mortality or Prolonged Length of Stay (Upper Quartile)	52.0 (22.2)	53.0 (22.6)	18.0 (23.1)	0.986
30-day readmission	46.0 (20.6)	47.0 (20.6)	7.0 (9.6)	0.084
One-year mortality	64.0 (27.4)	41.0 (17.5)	15.0 (19.2)	0.03*

SD, standard deviation.

* Post-hoc pairwise comparisons for 1-year mortality (Bonferroni-corrected): anemia vs. normocythemia, p = 0.044; anemia vs. polycythemia, p = 0.604; normocythemia vs. polycythemia, p = 1.000.

**Fig 2:**
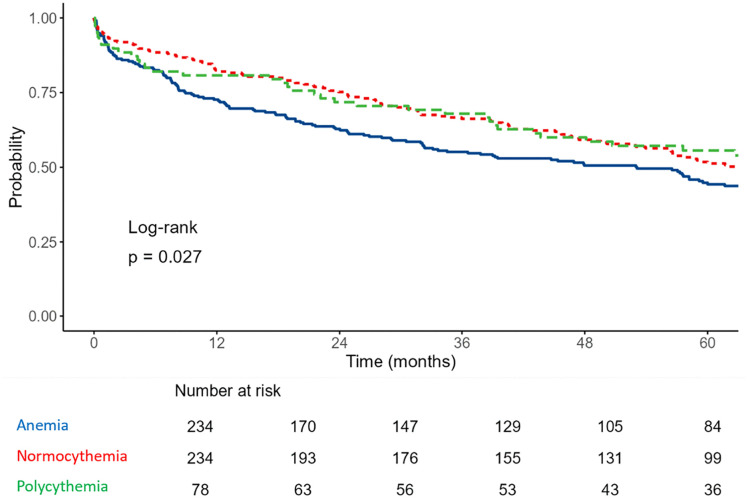
Kaplan–Meier survival curves for patients in the matched cohort (Mahalanobis distance matching, 3:3:1), stratified by hemoglobin group. The overall log-rank test showed a significant difference between groups (p = 0.027). In Cox proportional hazards analysis (reference = normocythemia), anemia was associated with higher mortality (HR 1.30, 95% CI 1.03–1.63, p = 0.026), whereas polycythemia was not (HR 0.90, 95% CI 0.64–1.27, p = 0.533). Post-hoc pairwise log-rank tests with Bonferroni correction were not statistically significant (anemia vs. normocythemia p = 0.073; anemia vs. polycythemia p = 0.109; normocythemia vs. polycythemia p = 1.000). Numbers at risk are displayed below the x-axis.

Kaplan–Meier survival analysis is shown in Fig 2. Survival differed significantly between groups (overall log-rank p = 0.027). In Cox proportional hazards analysis within the matched cohort (reference = normocythemia), anemia was associated with higher long-term mortality (HR 1.30, 95% CI 1.03–1.63, p = 0.026), whereas polycythemia was not (HR 0.90, 95% CI 0.64–1.27, p = 0.533). Post-hoc pairwise log-rank tests with Bonferroni correction did not demonstrate statistically significant differences (anemia vs. normocythemia p = 0.073; anemia vs. polycythemia p = 0.109; normocythemia vs. polycythemia p = 1.000). The survival curves for polycythemia and normocythemia overlapped, with the overall difference driven by the anemia group.

### Sensitivity analysis: Clinical outcomes – weighted cohort

**Fig 3:**
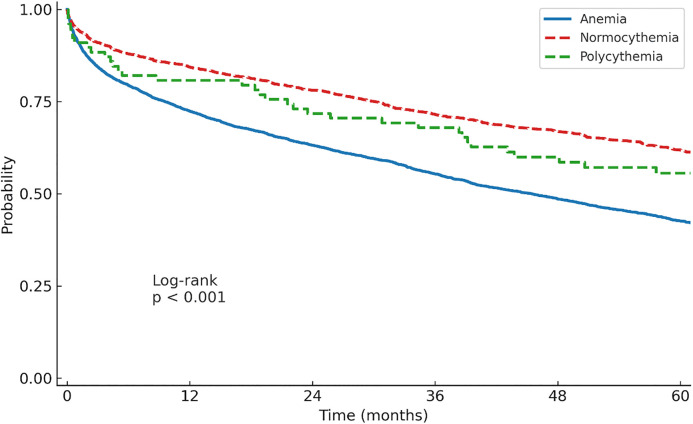
Kaplan–Meier survival curves from the sensitivity analysis using entropy balancing (EBAL), stratified by hemoglobin group. The overall weighted log-rank test was significant (p < 0.001). In weighted Cox proportional hazards analysis (reference = normocythemia), anemia was associated with higher mortality (HR 1.76, 95% CI 1.61–1.92, p < 0.001), whereas polycythemia was not (HR 1.14, 95% CI 0.83–1.57, p = 0.410). Post-hoc weighted log-rank tests with Bonferroni correction showed significant differences between anemia and normocythemia (p < 0.001) and between anemia and polycythemia (p = 0.015), but not between normocythemia and polycythemia (p = 0.931). Numbers at risk are not displayed because weighting alters group counts.

As mentioned, EBAL achieved excellent balancing (Fig 1). EBAL-weighted survival curves were generated using the full cohort (Fig 3). The overall weighted log-rank test was significant (p < 0.001). In weighted Cox proportional hazards analysis (reference = normocythemia), anemia was associated with substantially higher mortality (HR 1.76, 95% CI 1.61–1.92, p < 0.001), whereas polycythemia was not (HR 1.14, 95% CI 0.83–1.57, p = 0.410). Pairwise weighted log-rank comparisons with Bonferroni correction showed significant differences between anemia and normocythemia (p < 0.001) and between anemia and polycythemia (p = 0.015), but not between normocythemia and polycythemia (p = 0.931), consistent with the findings of the matched cohort analysis.

## Discussion

The present study examined the prognostic impact of polycythemia in patients hospitalized with ADHF. In this large single-center cohort, polycythemia was not associated with increased in-hospital, short-term, or long-term mortality. These findings indicate that, unlike anemia which consistently portends adverse prognosis, polycythemia does not serve as a negative prognostic marker in the setting of ADHF.

Several prior studies on HF populations have reported findings in line with the present analysis. For example, Omoomi et al. recently reported that in the Persian Registry Of Cardiovascular Disease/Heart Failure (PROVE-HF), 11.6% of 3,652 ADHF patients had polycythemia (defined by similar thresholds used in our study), and that polycythemia showed no association with increased short-term mortality (median follow-up 11.0 ± 7.3 months) [26]. Additionally, Miller et al. demonstrated in a chronic HF cohort of 110 patients, identified either at discharge after an index HF admission or from a dedicated HF clinic at the Mayo Clinic, that polycythemia – defined by a red blood cell mass (RCM) >10% above normal – was present in 42.7% of patients and was associated with improved 1-year event-free survival (mortality or readmission) [27]. Both studies support our finding that polycythemia is not associated with adverse prognosis, and the latter even suggests a favorable association. Our study extends this line of evidence by evaluating a much larger ADHF cohort, applying longer follow-up, and using robust statistical methods despite the relatively small polycythemia subgroup.

A striking difference, however, is the markedly higher prevalence of polycythemia reported in those studies compared with ours. The higher prevalence reported by Omoomi et al. may be partly explained by regional and altitude-related differences. Their registry was conducted in Isfahan, a city located approximately 1,500 meters above sea level. A study from another Iranian city at similar elevation, Shiraz, documented higher upper limits of normal hemoglobin (up to 18.4 g/dL in men) [28], supporting the possibility that altitude contributes to a larger proportion of patients exceeding conventional thresholds. This, in addition to a younger and more male-dominant case mix and the exclusion of in-hospital deaths in PROVE-HF, likely contributed to the higher prevalence observed. In Miller’s analysis, the use of RCM measurement – known to identify substantially more cases of polycythemia than hemoglobin thresholds, with nearly half of patients with increased RCM falling below WHO Hb thresholds in one series [29] – further explains the much larger prevalence. Moreover, Miller evaluated discharge profiles intended to reflect stable chronic HF, whereas our measurements were taken at admission, when hypervolemia and hemodilution may reduce apparent hemoglobin levels and thus lower the observed prevalence. Taken together, these population characteristics, methodological, and timing differences provide a plausible explanation for the substantially lower prevalence observed in our cohort.

Beyond ADHF, the impact of polycythemia on outcomes has been explored in other conditions, with some notable differences. In ACS, we have previously demonstrated that elevated hemoglobin portends worse long-term outcomes [12]. Despite the pathophysiological links between ACS and ADHF, which motivated the current research question, the absence of an adverse association in HF can be explained. First, in the pure ACS population, polycythemia was predominantly observed in younger patients and smokers, reflecting a healthier bone marrow responding to hypoxia. By contrast, our ADHF cohort (although including some ACS-driven cases) included older patients with multiple chronic diseases both are associated with the prevalence of anemia; in this setting, patients capable of mounting a polycythemic response likely represent those with preserved marrow function and lower chronic disease burden, offsetting any potential adverse effect of polycythemia. Second, a similar pattern was observed in our prior work on marital status, where an association present in ACS did not persist in ADHF [30]. There, we suggested that HF prognosis is shaped by a complex interplay of interacting factors, which can obscure associations that are clearer in ACS. The same consideration applies here.

Similar patterns have been described in other acute–on–chronic conditions. In a large COPD cohort (the SubPopulations and InteRmediate Outcome Measures in COPD Study, SPIROMICS), approximately 12% of patients had secondary polycythemia, and importantly, these patients experienced fewer severe exacerbations requiring hospitalization than their normocythemic peers [31]. Likewise, in COPD patients with chronic respiratory failure on long-term oxygen therapy, higher-than-normal hemoglobin levels were associated with better survival compared with both anemic and normocythemic patients [32]. Taken together, the prognostic impact of polycythemia appears to depend on the underlying clinical context: in relatively pure acute settings such as ACS it may play a negative role, whereas in acute–on–chronic states such as ADHF and COPD exacerbations it more likely reflects better baseline health and confers neutral or even favorable prognosis.

Another noticeable observation in our study was the proportion of females in the polycythemia group, which was markedly lower (25.6%) compared with nearly half of patients in the normocythemia and anemia groups (54.1% and 47.7%, respectively). Although caution is warranted in interpreting unadjusted findings, especially within a small subgroup – the male predominance in polycythemia is well recognized. This association is likely explained by three factors: (1) men have higher baseline hemoglobin levels due to androgen-driven stimulation of erythropoiesis [33]; (2) hypoxia-related exposures such as smoking, COPD, and obstructive sleep apnea are more common in men [34]; and (3) even in primary polycythemia vera, a male predominance has consistently been described [35].

Also notable, approximately 70% of patients with polycythemia were non-smokers, despite smoking being nearly twice as prevalent in the polycythemia group compared with anemia. In the general population, smoking is the predominant driver of secondary polycythemia [34]; the low proportion of smoking-related polycythemia in this cohort likely reflects the suppressive effect of chronic heart failure on erythropoiesis [36]. Chronic inflammation, renal impairment, and nutritional deficiency – all hallmarks of advanced heart failure – may attenuate the erythropoietic response to hypoxic stimuli such as smoking, preventing most smokers from reaching polycythemic thresholds. Consequently, polycythemia in ADHF may disproportionately arise from non-smoking etiologies, or may reflect the preserved physiologic reserve discussed above. However, the small size of the polycythemia group (n = 78) precluded formal subgroup analysis by smoking status.

This study applied two complementary approaches to address the combined problem of a small exposure group and substantial between-group covariate differences: MDM and EBAL. These methods were chosen over conventional multivariable regression, which remains the most common approach in observational studies, because matching and weighting are often superior when handling rare exposures [37]. With respect to MDM, although propensity score matching (PSM) is generally preferred in studies of rare conditions [14], the literature emphasizes that alternative distance-based strategies should be considered, as they may sometimes achieve superior covariate balance, particularly when combined with caliper restrictions or propensity-based approaches [38,39]. In our study, pure MDM without calipers yielded excellent covariate balance and was therefore retained as the primary analytic method. With respect to weighting approaches, inverse probability weighting (IPW) based on propensity scores is the most widely used technique; however, it can be sensitive to model misspecification and to the influence of extreme weights [40]. In contrast, EBAL formulates a constrained optimization problem that guarantees exact balance on prespecified covariates (including higher-order terms such as age squared). This property makes EBAL more robust than IPW, particularly in settings with small exposure groups where unstable weights may distort survival estimates [15]. Therefore, EBAL was chosen over IPW for our study. Both MDM and EBAL produced balanced pseudo-randomized cohorts, allowing univariate outcome comparisons that are methodologically equivalent to multivariable regression in the original cohort, and thereby obviating the need for additional covariate adjustment.

To further characterize the between-group differences, post-hoc pairwise comparisons with Bonferroni correction were performed for all analyses with significant overall tests. The key finding was consistent across all analyses: polycythemia and normocythemia did not differ significantly (p = 1.000 in the matched cohort, p = 0.931 in the EBAL-weighted analysis). However, the anemia comparisons behaved differently between approaches. In the matched cohort (n = 546), pairwise differences between anemia and the other groups did not retain significance after correction, despite a significant overall log-rank test (p = 0.027) and significant pairwise difference in 1-year mortality (p = 0.044). In the EBAL-weighted analysis (n = 8,332), these differences were significant (p < 0.001 and p = 0.015, respectively). This discrepancy reflects the well-recognized sensitivity of post-hoc pairwise testing to sample size and correction method, rather than a true inconsistency in findings [41]. The overall pattern – worse outcomes with anemia, comparable outcomes between polycythemia and normocythemia – was consistent across all analyses and is evident from both the survival curves and the outcome data.

This study has several limitations. First and foremost, the small size of the exposure group limits the strength of its findings. Although robust statistical methods were applied to mitigate this limitation, a group of this size inherently lacks the power to detect modest associations, and advanced methods may amplify rather than eliminate this shortcoming. Nevertheless, polycythemia in HF patients is rare, and this study applied best available practices to address that rarity. The concordance with prior HF reports and with observations from other medical fields further supports the validity of the findings despite this limitation. Second, the study was based on medical records and therefore subject to potential inaccuracies in documentation or data entry, which may have led to some degree of misclassification. Third, hemoglobin values were obtained only at admission and may have been influenced by hypervolemia and hemodilution, potentially decreasing the size of the polycythemia group. It also precluded assessment of longitudinal trends. Fourth, Smoking status was not included in the matching model; while its prognostic role in hospitalized heart failure remains uncertain, residual confounding by smoking cannot be excluded. Fifth, obstructive sleep apnea was not captured in the dataset, and echocardiographic data were available for only 30.9% of the cohort, precluding classification by heart failure phenotype (HFrEF vs. HFpEF) or reliable assessment of guideline-directed medical therapy. Sixth, data on medications that may influence hemoglobin levels, such as erythropoiesis-stimulating agents and SGLT2 inhibitors, were not available in the dataset; nevertheless, their contribution to polycythemia in this cohort is likely minimal, as ESAs are used to correct anemia rather than induce polycythemia, and SGLT2 inhibitor use was limited during the study period with only a modest effect on hemoglobin levels. Seventh, the study spans a decade (2007–2017) during which heart failure management evolved; however, we have previously demonstrated in this same cohort that outcomes did not significantly change between the early and recent periods [42], and the matching and weighting procedures further mitigate the impact of any temporal trends by balancing covariates across groups within the same timeframe. Finally, although MDM and EBAL were applied to account for measured covariates, residual confounding by other unmeasured factors cannot be excluded.

## Conclusion

In conclusion, polycythemia is uncommon in patients hospitalized with ADHF and is not associated with adverse outcomes. These findings are consistent with prior reports of HF and acute exacerbation of chronic disease, and indicate that polycythemia does not represent a negative prognostic marker in such settings.
